# Seroprevalence of Hepatitis E Virus Among Schistosomiasis mansoni Patients Residing in Endemic Zone in Brazil

**DOI:** 10.3390/tropicalmed9120310

**Published:** 2024-12-20

**Authors:** Cristiane Tiburtino de Oliveira Gomes, Carolline Araujo Mariz, Andrea Dória Batista, Clarice Neuenschwander Lins de Morais, Lílian Araújo, Ana Virgínia Matos Sá Barreto, Michele Soares Gomes-Gouvêa, Ana Lúcia Domingues, Edmundo Pessoa Lopes

**Affiliations:** 1 Postgraduate Program in Tropical Medicine, Center of Medical Sciences, Universidade Federal de Pernambuco (UFPE), Recife 50670-420, PE, Brazil; alcdomingues@hotmail.com; 2 Department of Parasitology, Aggeu Magalhães Institute, Fiocruz, Recife 50740-465, PE, Brazil; carolline.mariz@fiocruz.br; 3 Faculdade de Medicina de Olinda (FMO), Olinda 53030-030, PE, Brazil; 4 Gastroenterology Division, Hospital das Clínicas/EBSERH, Universidade Federal de Pernambuco (UFPE), Recife 50670-901, PE, Brazil; andreadoriabatista@gmail.com (A.D.B.); maia_lilian@hotmail.com (L.A.); 5 Department of Internal Medicine, Center of Medical Sciences, Universidade Federal de Pernambuco (UFPE), Recife 50670-901, PE, Brazil; 6 Department of Virology, Aggeu Magalhães Institute, Fiocruz, Recife 50740-465, PE, Brazil; clarice.lins@fiocruz.br; 7 Department of Health, Maurício de Nassau University Center, Recife 51021-140, PE, Brazil; aninhabarretoo@gmail.com; 8 Laboratory of Gastroenterology and Tropical Hepatology (LIM-07), Institute of Tropical Medicine, Faculdade de Medicina da Universidade de São Paulo, São Paulo 05403-000, SP, Brazil; gomesmic@yahoo.com.br

**Keywords:** anti-HEV, coinfection, hepatic fibrosis, hepatitis E virus, Schistosomiasis mansoni

## Abstract

The occurrence of hepatitis E virus (HEV) in patients with Schistosomiasis mansoni (SM) is still poorly understood in Brazil. The objective of this study was to estimate the seroprevalence of anti-HEV IgG in patients with SM and its association with the periportal fibrosis (PPF), assessed by serum markers and ultrasound criteria. This cross-sectional study was carried out in an endemic area in Pernambuco, Brazil, with schistosomal patients who underwent coproscopic survey. Anti-HEV antibody IgG were evaluated by using ELISA (Euroimmun^®,^ Lübeck, Germmany). In positive cases, HEV-RNA was tested by using real-time PCR. Among the 286 patients (60.8% women; 56% 18–44 years), 116 (40.6%) had advanced PPF (Niamey pattern D/E/F). Anti-HEV IgG was positive in 15 (5.24%), and all were HEV-RNA negative. Anti-HEV IgG was more frequent in patients with an advanced PPF (D/E/F) pattern (*p* = 0.034) and those with the largest spleen diameter (*p* = 0.039). In this study, the occurrence of anti-HEV IgG in patients with SM was higher than described in the same region and more frequent among patients with evidence of advanced liver fibrosis.

## 1. Introduction

Hepatitis E virus (HEV) infection is a relevant public health problem as it is endemic in most industrialized countries [1]. The World Health Organization (2023) estimates that 20 million people are infected globally per year, with 3.3 million symptomatic cases [2].

HEV belongs to the family Hepeviridae, which is divided into two subfamilies. Subfamily Orthohepevirinae members infect mammals and birds, and subfamily Parahepevirinae members infect trout and salmon. The subfamily Orthohepevirinae is divided into four genera, with members of the genus Paslahepevirus having a different host range, being found in humans as well as in a wide range of domestic and wild mammals (pig, wild boar, cow, deer, rabbit, camel) [3,4].

Members of the species *Paslahepevirus balayani* have been assigned to eight different genotypes (HEV-1 to HEV-8), of which four (HEV-1, HEV-2, HEV-3 and HEV-4) are most associated with HEV infection in humans. HEV 1 and HEV 2 genotypes occur mainly in places with little health infrastructure in developing countries, such as Africa, Asia, Central America, and the Middle East [3,4,5].

Transmission occurs via the fecal–oral route through water contaminated by human feces, and contamination from person to person is infrequent. Parenteral and vertical transmissions have also been described [1,4,5].

HEV 3 and HEV 4 genotypes occur mainly in developed countries, such as the United States of America (USA), Japan, and China [6]. The infection displays zoonotic transmission through contact with infected animals, such as pigs, rabbits, sheep, cats, dogs, and goats, or through the consumption of food, such as raw or undercooked meat or fruits and vegetables washed with contaminated water. HEV 3 has already been detected in mussels and oysters, as well as through parenteral transmission [1,4,7].

Although HEV infection is self-limited, patients with both chronic liver disease (CLD) and HEV have a higher risk of clinical decompensation and worse prognosis [1,4,8]. In immunosuppressed patients, HEV infection can become chronic, with persistent liver aggression, fibrosis, and cirrhosis [1,4,5].

Schistosomiasis mansoni (SM) is a persistent health problem in Brazil, affecting around 1.5 million people. It is among several neglected tropical diseases that affect low-income populations worldwide [9].

According to data from the Brazilian Public Unified Health System department (DATASUS), 909 cases of schistosomiasis were confirmed in 2023 in Brazil, of which 299 were in the Northeastern region, with 50 cases in the state of Pernambuco [10].

SM and HEV infections can both be acquired in places with precarious sanitary conditions. In endemic regions, patients with advanced forms of SM, such as hepatosplenic SM, may progress more quickly to cirrhosis when associated with viral hepatitis [11].

There is some evidence of HEV circulation in Brazil [12]. A study published by Oliveira et al. (2023) describes a high prevalence of HEV 3 in pigs, both on large farms and on family farms, and this genotype is the only one identified in Brazil, presenting as zoonotic transmission [13].

Only two studies have been published on the prevalence of HEV infection with small sample sizes, which involved SM patients in Brazil—one in Bahia and the other in Pernambuco. In both, the patients were evaluated in hepatology reference services [14,15].

Given the lack of data on the occurrence of HEV in patients with SM living in endemic areas in Brazil, this study aims to estimate the seroprevalence of anti-HEV IgG in these patients. Considering that this co-infection may predispose patients to a worse outcome, the pattern of periportal fibrosis (PPF) was also investigated through serum markers and ultrasound criteria.

## 2. Materials and Methods

### 2.1. Study Design, Period, Location, and Population

This cross-sectional study used a database and serums of schistosomiasis patients, who participated in the validation study of the Coutinho Index, carried out in Jaboatão dos Guararapes, between 2015 and 2016. The Coutinho Index uses alkaline phosphatase (ALP) and platelet number as a tool to distinguish patients without or with little PPF from those with advanced PPF [16].

The municipality of Jaboatão dos Guararapes is in the Metropolitan Region of Recife, an endemic area for SM. With a territorial area of 258,724 km^2^, it is the second most populous city in the state of Pernambuco in Northeastern Brazil, according to the 2022 census [17]. The Schistosomiasis Control Program in the municipality is linked to primary health care and actively searches for cases through coprological examination [18].

The previous study included 379 individuals (age ≥ 18 years) who reported river bathing and had *Schistosoma mansoni* eggs in the Kato–Katz stool test. Those reporting diagnoses with liver disease from other etiologies, including hepatitis B or hepatitis C; alcohol intake above >210 g/week for men or 140 g/week for women; use of hepatotoxic drugs; liver transplant recipients; chronic kidney disease; or those with splenectomy were excluded [16].

### 2.2. Clinical and Laboratory Evaluation

Patients were interviewed to collect demographic data and underwent clinical evaluation. Subsequently, they underwent ultrasound (GE Healthcare Logiq E) examinations of the upper abdomen, performed by the same experienced operator (ALCD). To categorize the PPF pattern, the Niamey classification was used, which identifies six fibrosis patterns: A—absence of fibrosis; B—doubtful fibrosis; C—peripheral fibrosis; D—central fibrosis; E—advanced fibrosis; F—very advanced fibrosis [19].

After the ultrasound scan, 10 mL of blood was collected in a peripheral vein for ALP measurement, platelet count, HBsAg (HBsAg rapid test VIKIA^®^, Marcy l’Étoile, France), and anti-HCV (WAMA Diagnostic IMMUNO-RAPID test, São Carlos, Brazil). The remainder of the samples were centrifuged, and the serum was frozen. ALP dosing was performed using an ALP kit from Labtest (Singapore). Serum levels were expressed as a ratio of the value obtained by the upper limit of normal (ULN). Platelet count was performed using ABX Micros ES60 equipment from Horiba (Kyoto, Japan). With the serum levels of ALP and platelet count results, the Coutinho Index was calculated using the following formula: [(ALP/ULN)/(platelets)] × 100 [16].

### 2.3. Anti-HEV IgG Research

Stored serum samples, frozen at −20 °C in a freezer in the laboratory of the Department of Virology of the Aggeu Magalhães Institute, Fiocruz, Recife, Pernambuco, Brazil, were thawed and tested for anti-HEV IgG by employing the ELISA method, using the commercial hepatitis E (HEV) IgG kit (Euroimmun^®^, Lübeck, Germany). Indeterminate results were considered anti-HEV negative.

### 2.4. HEV-RNA Research

In positive cases, HEV-RNA was investigated by using real-time quantitative reverse transcription polymerase chain reaction (qRT-PCR), extracted from 200 μL of serum using the QIAmp^®^ MinElute^®^ Virus Spin Kit (QIAGEN, Hilden, Germany), according to the manufacturer’s instructions. Viral RNA was eluted in 60 μL of elution buffer, and 5 μL was used for amplification by one-step real-time PCR using the kit QuantiFast Pathogen RT-PCR + IC (QIAGEN, Hilden, Germany) and primers and a TaqMan probe, described previously to target the highly conserved ORF3 region [20]. These procedures were carried out in the in the LIM/52 Virology Laboratory of Tropical Gastroenterology and Hepatology, Institute of Tropical Medicine at the University of São Paulo, Brazil.

### 2.5. Study Ethics

The research was approved by the Ethics and Research Committee of the Clinical Hospital, Federal University of Pernambuco—HC/UFPEE, under opinion no. 6.470.362.

### 2.6. Statistical Analysis

The data were taken from a database used in the previous study and stored in a database created exclusively for this research. For the descriptive analysis, absolute frequency and percentages were calculated. The prevalence of HEV infection and its respective 95% confidence interval (95% CI) were calculated. Pearson’s chi-square test and, when necessary, Fisher’s exact test was used to verify the statistical significance differences found in the frequency distribution of the variables, according to the positivity of the serological marker IgG for HEV. Results with a *p*-value < 0.05 were considered statistically significant. Data were analyzed using the Stata Program, version 15 (StataCorp, College Station, TX, USA).

## 3. Results

Among the 379 aliquots of serum from the previous study, we excluded 5 (1.32%) that were positive for HBsAg, 1 (0.26%) that had anti-HCV, and 87 (22.90%) that presented insufficient material for the research of anti-HEV IgG and HEV-RNA. Hence, 286 samples were evaluated in this study.

The sociodemographic and laboratory characteristics of the 286 patients with SM are described in Table 1. The ultrasound characteristics presented in Table 2 include the evaluation of the periportal fibrosis pattern, according to the Niamey classification, and the spleen size.

Anti-HEV IgG was positive in 15 of 286 patients [5.24%; 95% CI: 3.17–8.54)], and all HEV-RNA tests were negative. The serum levels of ALP (*p* < 0.001) and the values of the Coutinho Index (*p* < 0.001) were significantly higher in these 15 positive patients. Also, the platelet count tended to be lower (*p* = 0.067) in cases that were anti-HEV IgG positive (Table 1).

Patients were divided into two groups according to the Niamey PPF classification: those with absent or mild PPF (A/B/C patterns) and those with moderate and advanced PPF (D/E/F patterns). A higher occurrence of anti-HEV IgG was observed in patients with more advanced PPF patterns (*p* = 0.034). Additionally, the spleen was longer (*p* = 0.039) in cases with positive anti-HEV IgG (Table 2).

## 4. Discussion

Among hepatotropic viruses, HEV infection is still little studied. However, there is growing interest in understanding the impact of this agent when infecting patients with CLD or immunosuppression. In Brazil, data on the repercussions of PPF-associated HEV infection in SM patients and its consequences are very scarce. One of the reasons for the scarcity could be the low availability of tests for anti-HEV research, especially for patients from the Brazilian Public Unified Health System.

In the present study, 5.24% of the 286 SM patients residing in an endemic area tested positive for anti-HEV IgG, and all tested negative for HEV-RNA. A meta-analysis study found a global prevalence of anti-HEV IgG of 12.47%, with the following distribution: Africa (21.76%), Asia (15.80%), Europe (9.31%), North America (8.05%), South America (7.28%), and Oceania (5.99%). A sub-analysis of ten Brazilian studies, with about 4739 cases, demonstrated a 6.39% prevalence of the IgG antibody against HEV [21].

Another systematic review and meta-analysis demonstrated a higher prevalence of anti-HEV IgG in serum in the USA than in Latin America, regardless of the patient cohort, test used, or methodological quality (OR: 1.82 [1.06–3.08], *p* = 0.03). The prevalence found in the USA (9%) was twice that observed in Brazil (4.2%) using the same test. In patients who are immunocompromised patients (HIV-infected or solid organ transplanted), people at occupational risk (direct contact with pigs or wild animals or forest workers), and those with chronic diseases (liver or kidney, and patients on dialysis) in the USA, the rates were 12.2%, 12.1%, and 10.4%, respectively. For the same profile of patients in Brazil, the rates found were 5.8%, 5.7%, and 4.9%, respectively [22].

In Brazil, a systematic review and meta-analysis revealed a prevalence of 6% in the adult population with anti-HEV IgG, 3% in the general population, and 7% in blood donors [12]. The prevalence of HEV infection varies between regions of Brazil and is highest in the southern region. In fact, a study in Rio Grande do Sul with 3000 blood samples found anti-HEV IgG in 59.4% of the individuals tested. The authors suggest that this high prevalence may be related to the higher consumption of undercooked meats in their region. They also draw attention to the use of pig manure in fertilizers, which is a risk factor for the HEV contamination of vegetables and water [23].

In Pernambuco, the described prevalence of anti-HEV is lower. Research conducted with 996 blood donors in Recife found 0.9% seroprevalence for anti-HEV IgG [24]. In the present study, the higher prevalence of anti-HEV IgG in SM may be related to the place of residence, since the patients in this study residing in a Schistosomiasis endemic area possibly had greater contact with waters contaminated by manure and with domestic animals, in addition to the precarious sanitary infrastructure in this region.

Few data are available on the co-infection of anti-HEV and schistosomiasis in Northeastern of Brazil. Studies in Bahia, with 30 patients, and in Pernambuco, with 80 patients, observed prevalences of 10% and 18.8%, respectively [14,15]. In the second study that evaluated schistosomiasis in Recife, the authors suggested that the high prevalence may be due to the patients’ precarious socioeconomic conditions, leading to the acquisition of both infections. Note that patients in both studies were selected in specialized outpatient clinics of tertiary hospitals and had more severe forms of SM [14,15].

In the study in Pernambuco, cases with positive anti-HEV also had higher levels of liver enzymes [15]. On the other hand, the present study evaluated patients in an endemic zone with less advanced forms of SM.

Some authors have already demonstrated that HEV infection in immunosuppressed patients predisposes progression to chronic hepatitis and faster progression to cirrhosis [25,26,27,28]. A study with 85 HEV-infected solid organ transplant patients in 17 centers in the USA and Europe observed that 56 (65.9%) cases evolved to chronic hepatitis, of which 8 developed cirrhosis and 2 required a second liver transplant [28]. Nevertheless, a Brazilian study involving 294 liver transplant recipients did not identify a higher degree of fibrosis in patients presenting HEV infection markers, including both antibodies and HEV-RNA [29].

Conversely, unfavorable evolution was observed in a Brazilian study that evaluated 618 patients chronically infected with HEV, whose prevalence of anti-HEV IgG in cirrhotic patients was higher than in non-cirrhotic patients (13.2% vs. 8%, OR = 1.74, *p* = 0.04). The authors suggested that the association between cirrhosis and previous HEV infection may accelerate the process of liver fibrosis in patients with chronic hepatitis C [30].

In Bahia, 301 patients with CLD of various etiologies were investigated, and a 12.95% prevalence of anti-HEV IgG was observed [31]. Moreover, Araújo et al. (2023), also in Brazil, investigated anti-HEV IgG in 227 patients with CLD of various etiologies, including SM, and observed 7 positive cases (3.08%). They found a higher frequency of anti-HEV among those with evidence of more advanced liver disease, including higher AST to Platelet Ratio Index (APRI) and Fibrosis-4 (FIB-4) Index for Liver Fibrosis. Among the seven cases with positive anti-HEV, four had SM in the etiology of liver disease, one with the SM isolated form, and three had SM associated with other causes of CLD [32].

In the present study, a higher frequency of anti-HEV IgG was also observed among cases with SM and evidence of more advanced PPF, both through the fibrosis pattern found in ultrasound and Coutinho Index, corroborating the data described above in patients with CLD of other etiologies [15,30,32].

Finally, the main limitation of this study was certainly its design, a cross-sectional study. The higher frequency of anti-HEV IgG in the most severe cases does not allow us to establish whether HEV co-infection led to the evolution to more advanced forms of SM or whether it was due to greater exposure to HEV contamination in outpatient clinics or during medical examinations.

## 5. Conclusions

In this study, the seroprevalence of anti-HEV IgG in patients with schistosomiasis residing in an endemic zone of Northeastern Brazil was higher than that described in the same region and more frequent among patients with evidence of more advanced liver fibrosis. Prospective follow-up studies with a cohort of patients that have schistosomiasis will be needed to clarify whether HEV worsens the evolution of helminthiasis or whether patients with the more advanced forms of SM are more exposed to HEV contamination.

## Figures and Tables

**Table 1:** Demographic and laboratory characteristics of the 286 patients with Schistosomiasis mansoni in an endemic area, according to anti-HEV IgG serology status, Pernambuco, Brazil.

Characteristics	Total	Anti-HEV IgG		*p*-Value
		Positive	Negative	
Sex (n/%)				
Male	112 (39)	8 (7.1)	104 (92.8)	0.248 ^a^
Female	174 (61)	7 (4.0)	167 (96.0)	
Age (years) (n/%)				
18–44 (%)	160 (56)	8 (5.0)	152 (95.0)	0.844 ^a^
45–80 (%)	126 (44)	7 (5.5)	119 (94.5)	
Platelets(/mm^3^) *	260,244 ± 100.5	214,000 ± 93.5	262,804 ± 100.4	0.067 ^b^
ALP (IU/L) *	74.7 ± 28.3	102.5 ± 80.2	73.2 ± 21.50	<0.001 ^b^
Coutinho Index *	0.40 ± 0.90	1.37 ± 3.70	0.35 ± 0.20	<0.001 ^b^

^a^—Chi-square test; ^b^—*t*-test; %: percentage; n, number; ALP: alkaline phosphatase. * Mean values ± SD.

**Table 2:** Evaluation of the periportal fibrosis pattern, according to the Niamey classification, and spleen size of the 286 patients with Schistosomiasis mansoni in an endemic zone, according to anti-HEV IgG serology status, Pernambuco, Brazil.

Characteristics	Total	Anti-HEV IgG		*p*-Value
		Positive	Negative	
Periportal fibrosis (n/%)				
Patterns A/B/C	170 (59.4)	5 (2.9)	165 (97.1)	0.034 ^a^
Patterns D/E/F	116 (40.6)	10 (8.6)	106 (91.4)	
Spleen (cm) *	9.65 ± 2.1	10.70 ± 4.0	9.59 ± 2.0	0.039 ^b^

^a^—Fisher’s exact test; ^b^—*t*-test; %: percentage; n, number. * Mean values ± SD.

## Data Availability

The database used in this study is available on request from the corresponding author.
